# Nanotechnology Platform for Advancing Vaccine Development against the COVID-19 Virus

**DOI:** 10.3390/diseases11040177

**Published:** 2023-12-10

**Authors:** Nusrat Chowdhury, Anup Kundu

**Affiliations:** Department of Biology, Xavier University of Louisiana, New Orleans, LA 70125, USA; chowdhury_nusrat@ymail.com

**Keywords:** liposomes, COVID-19, nanoparticle, adjuvant, mRNA vaccine, virus, nanotechnology, advances

## Abstract

The COVID-19 pandemic has had a profound impact on societies, public health, healthcare systems, and the world economy. With over 771 million people infected worldwide and a staggering death toll exceeding 6,960,783 as of 4 October 2023 (according to the World Health Organization), the urgency for a solution was paramount. Since the outbreak, the demand for immediate treatment for COVID-19 viral infection, as well as for effective vaccination against this virus, was soaring, which led scientists, pharmaceutical/biotech companies, government health agencies, etc., to think about a treatment strategy that could control and minimize this outbreak as soon as possible. Vaccination emerged as the most effective strategy to combat this infectious disease. For vaccination strategies, any conventional vaccine approach using attenuated live or inactivated/engineered virus, as well as other approaches, typically requires years of research and assessment. However, the urgency of the situation promoted a faster and more effective approach to vaccine development against COVID-19. The role of nanotechnology in designing, manufacturing, boosting, and delivering vaccines to the host to counter this virus was unquestionably valued and assessed. Several nanoformulations are discussed here in terms of their composition, physical properties, credibility, and applications in past vaccine development (as well as the possibility of using those used in previous applications for the generation of the COVID-19 vaccine). Controlling and eliminating the spread of the virus and preventing future recurrence requires a safe, tolerable, and effective vaccine strategy. In this review, we discuss the potential of nanoformulations as the basis for an effective vaccine strategy against COVID-19.

## 1. Introduction

**Figure 1:**
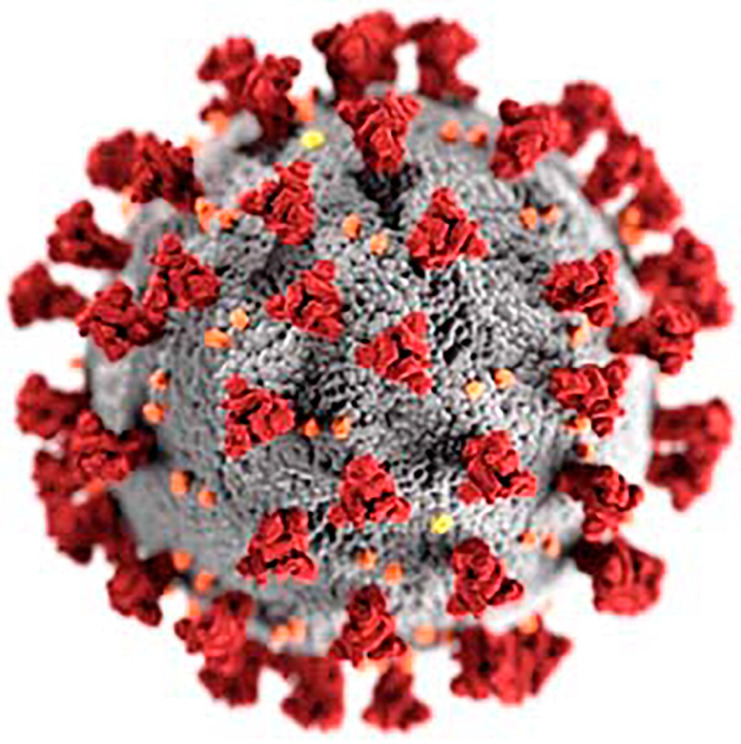
A graphical representation of the structure of coronavirus (SARS-CoV-2). Source: Centers for Disease Control and Prevention—Public Health Image Library. Credit: Alissa Eckert, MS, Dan Higgins, MAM.

In December 2019, a coronavirus outbreak was first made public in China and reported to have over 7000 infected patients and 170 dead. The new virus was named SARS-COVID-19 (SARS-CoV-2). The name comes from Coronavirus Disease 2019. COVID-19 eventually spread globally and was declared a global pandemic in March 2020 [1]. According to the World Health Organization (WHO), as of 4 October 2023, over 771 million people have been infected with COVID-19, and the total death toll has risen to over 6,960,783. Among the countries that were affected the greatest are the United States of America, India, Brazil, France, Germany, Italy, and the United Kingdom. As of 4 October 2023 in the USA, more than 103,436,000 confirmed cases of COVID-19 and more than 1,127,000 deaths have been reported to the WHO. The common symptoms of this infection are fever, fatigue, dry cough, shortness of breath, muscle or body aches, headache, etc. It was later discovered that the severe acute respiratory syndrome (SARS)-like disease emerged from an open market in Wuhan, China, and was triggered by a novel type of coronavirus. There are six previously identified coronavirus infections: 229E and NL63 (alpha coronavirus); OC43 and HKU1 (beta coronavirus); SARS-CoV (beta coronavirus—causes Severe Acute Respiratory Syndrome); and MERS-CoV (beta coronavirus—causes Middle East Respiratory Syndrome). This new coronavirus can infect both the lower and upper respiratory tracts, and sometimes the infection can even reach down to the alveoli. In children, COVID-19 has been shown to cause severe inflammation in multiple parts of the body, although the symptoms are mild for most children. A graphical representation of the structure of this type of coronavirus (SARS-CoV-2) is shown in Figure 1. Currently, the most commonly available COVID-19 vaccines are the Comirnaty and Pfizer-BioNTech COVID-19 Vaccine and the Spikevax and Moderna COVID-19 Vaccine. As the knowledge on COVID-19 is advancing, the need for new therapeutics, like monoclonal antibodies and advanced vaccines, might be necessary in the near future.

Vaccination is the most effective way to fighting any infectious disease. Many viral diseases like influenza, yellow fever, measles, tetanus, diphtheria, smallpox, and polio can be currently controlled only because of the presence of specific vaccines available in the market. But there are still some viral diseases, like HIV, etc., that require improvements in their treatment to have them completely eradicated or at least manageable, and COVID-19 is a new addition to this group. Conventional vaccines use subunit protein antigens, inert pathogens (inactivated), or attenuated viruses to stimulate a specific immune response. These vaccines often cause safety concerns for the elderly and immune-compromised patients. Certain vaccines also need additional adjuvants to boost immune responses since they elicit weaker responses [2,3]. On top of that, the high genetic mutations of certain viruses (i.e., influenza virus) reduce the effectiveness of the vaccines [4]. Hence, there is a need for a new generation of vaccines that are safer, cause less or no unwanted side effects, and provide high efficacy.

**Figure 2:**
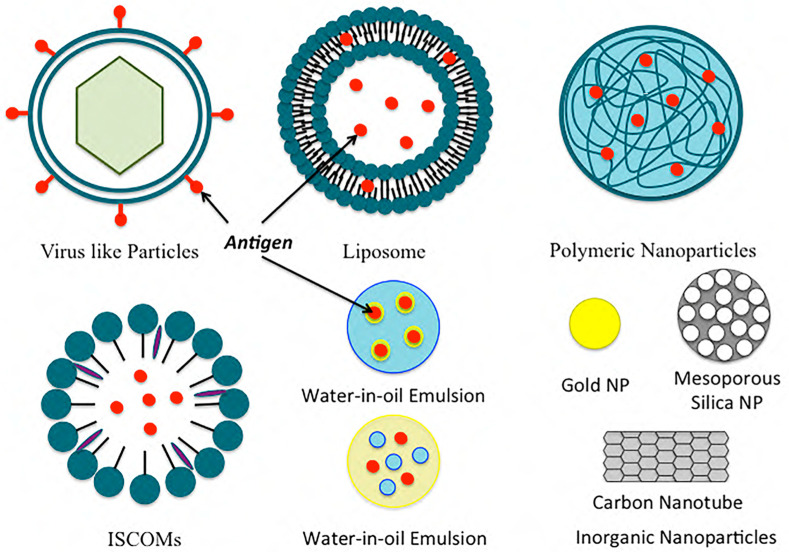
Examples of common nanoformulations used for vaccine delivery.

In the formulation of COVID-19 vaccines and their efficient delivery to the host, a nanotechnology platform can prove to be useful over the conventional techniques of vaccine development. The wider application of nanotechnology lies in the capability of making smaller particles in the range of 1 nm to a couple hundred nanometers. Nanoparticle formulation can be used as a delivery system to improve the delivery of antigens by acting as an antigen depot, by targeting APCs, or by acting as an immune–stimulatory adjuvant to induce protective immunity. Due to their smaller size, nanoparticles engulfed into cells via endocytosis, phagocytosis, and micropinocytosis can elicit appropriate responses and immunogenicity. They can improve the antigenicity of antigens (adsorbed/conjugated) by acting as adjuvants or as antigens themselves. Nanoparticle formulations can also induce innate and adaptive immune responses. They can extend the half-life of many vaccines and act as immune potentiators. They can help in the controlled release of antigens. Some of the common nanoformulations such as polymeric nanoparticles, inorganic nanoparticles, liposomes, virosomes, ISCOM, and others are discussed here in terms of their composition, physical properties, credibility, and applications in past vaccine development (as well as the possibility of using those used in previous applications for the generation of the COVID-19 vaccine) (Table 1). A brief illustration of these particles is also shown in Figure 2. Controlling and eliminating the spread of the coronavirus and preventing future recurrence requires a safe, tolerable, and effective vaccine strategy. In this review, we discuss the potential of nanoformulations as the basis for an effective vaccine strategy against COVID-19.

## 2. Nanoparticle Vaccine Adjuvants and Delivery Systems

### 2.1. Polymeric Nanoparticles

Polymeric nanoparticles can be divided into two categories: natural polymeric nanoparticles and synthetic polymeric nanoparticles. Two of the most widely used natural polymeric NPs in pharmaceuticals and medical fields are chitosan and alginate. Chitosan is derived from chitin, and it is biocompatible, biodegradable, and non-toxic. It can be easily fabricated into different shapes and sizes. Mehrabi et al. designed mannosylated chitosan (MC) nanoparticles for targeting hepatitis B virus surface antigen (rHBsAg) [14]. The nanoparticles showed an extended release for over 49 days and were successful in producing immunogenicity against the virus [14]. Alginate is an anionic polysaccharide derived from marine brown algae cell walls. It is a natural, biodegradable, and non-toxic mucoadhesive polymer [53,54]. Sarei et al. immunized guinea pigs with diphtheria toxoid-loaded alginate nanoparticles in vivo and found that the NPs produced better humoral immune responses than the conventional vaccine [17]. Hyaluronic acid (HA), another type of natural polymer made of N-acetyl-D-glucosamine and D-glucuronic acid, can bind with several cell surface receptors, such as TLR4, TLR2, and CD44, thus leading to many physiological activities [55,56]. As HA can activate TLRs/CD44 on immune cells, it is being investigated in cancer therapy.

Polylactide (PLA), polycaprolactone (PCL), and poly (d,l-lactic-coglycolic acid) (PLGA) are some of the most studied synthetic polymers used in the preparation of oral, mucosal, and systemic vaccine formulations. Among them, PLGA has been approved by both the European Medicine Agency and the Food and Drug Administration (FDA). PLGA NPs can be used individually or combined with natural polymers for vaccine delivery. Gu et al. created an immunopotentiator along with a protein antigen in PLGA. The resulting NP improved the ratio of CD4+ to CD8+ T cells, thus inducing a strong and continuous cellular immune response [57]. On the contrary, polyglycolic acid (PGA) is highly crystalline and has a slow degradation property that limits its use as a vaccine delivery system.

### 2.2. Inorganic Nanoparticles

Inorganic NPs can be used as adjuvants or delivery vehicles for antigens to improve immune responses (Table 1). Due to their rigid structure and easier synthesis, they are also being considered for pharmaceutical formulation preparations and applications. They are mostly non-biodegradable. Some of the commonly used inorganic NPs are gold, carbon, silica, aluminum-based, calcium phosphate, and magnetic NPs. Gold NPs can be formulated into different shapes (such as rods, spheres, cubes, and layers) and different sizes. They have been used for vaccination against influenza and HIV viruses and as a delivery vehicle for proteins and peptides. Niikura et al. studied the effects of several gold NPs by varying their shapes and sizes and concluded that the different sizes of gold NPs activate the immune system through different cytokine pathways [20]. Carbon nanoparticles are inorganic nanoparticles that can be modified into mesoporous spheres and nanotubes. Carbon nanotubes can give different levels of responses when conjugated to peptide and protein antigens. They are also studied for the oral delivery of vaccines [21]. Silica-based nanoparticles are adjuvants for the effective induction of adaptive immune responses. In the studies by An et al., surface-loaded amorphous silica NPs were used for lymph node targeting, and improved B and T cell immune responses were found when compared to soluble vaccines [58]. Mesoporous silica NPs (MSNs) have been proven to be excellent candidates for drug and gene delivery. MSNs improve leakage- and instability-related issues that are common with other type of nanoparticles [59,60,61,62]. Jimenez-Perianez et al. have used mesoporous silicon microparticles (MSMPs) to deliver specific class I-restricted T cell epitopes to human monocyte-derived dendritic cells (MDDCs), which generated an effective antiviral cytotoxic T lymphocyte (CTL) response [63]. Aluminum hydroxide and several aluminum salts, known as alums, are also inorganic NPs that have been used as adjuvants in animal vaccines, as well as in human vaccines. Alums can enhance antigen-specific immune responses. The efficacy of alums also depends on their shape; for example, rod-shaped alums show stronger dendritic cell responses than sphere-shaped alums [64]. CaP NPs have been used against the flu, HBV, and anthrax, as well as for the delivery of DNA vaccines. CaP NPs are promising candidates for mucosal adjuvants. Magnetic NPs are also inorganic NPs that have been approved by the FDA for vaccine delivery.

### 2.3. Liposomes

Gregoriadis and Allison reported liposomes as an inducer of immune responses to the entrapped or associated antigens in 1974 [65]. Since then, liposomes and liposome-derived nanovesicles (archaeosomes and virosomes) have sparked interest in the development of vaccines. Liposomes can enhance drug solubility, lower dose-limiting toxicities, and minimize unwanted side effects. Liposomes are easy to prepare. They are versatile, and the lipid composition can be altered to obtain a desirable size, charge, and entrapment of antigens or adjuvants. Liposomes are capable of entrapping both water-soluble compounds, like proteins, peptides, and nucleic acids, as well as lipophilic compounds like antigens, adjuvants, and linker molecules. The liposomes can also be labeled with different targeting moieties for their targeted delivery to the desired cells and tissues. Liposomal vaccines are usually intramuscular or subcutaneous. Studies have shown that the different sizes of liposomes give different levels of responses for the same injection site [66]; another study has shown that cationic liposomes have no differences in the release of antigen, but they do affect the concentration of the antigens in the regional lymph nodes [67,68]. Kaur et al. studied pegylated cationic liposomes and found that the pegylation of liposomes altered the immune responses due to the reduction in the depot effect [29]. The studies by Badiee et al. showed that larger particles have better lymphatic drainage [69]. Neutral liposomes have also been extensively studied. Moon et al. formulated multilamellar vesicles entrapping immune-stimulatory molecules in the bilayers and antigens within the core of a liposome. The resulting liposomes elicited strong T cell and antibody responses [27]. Archaeosomes are another type of stable liposomes composed of natural lipids extracted from archaea or synthetic archaeal lipids [70]. Patel et al. studied archaeosomes that were prepared from polar lipids [71]. They prepared a trivalent vaccine, a univalent archaeosome vaccine, and an admixture vaccine. The vaccines were given subcutaneously, and the specific IgG1 and IgG2a responses were checked after 112 days. Their study showed that the trivalent and admixture vaccines had strong specific antibody responses to all three antigens used in the preparation, and it was comparable to the ones induced in the control mice administered with univalent vaccines. Liposomal formulations, such as Doxil^®^, have been approved by the Food and Drug Administration. Stimuvax^®^, also known as L-BLP25, by Merck and Biomira is another liposomal vaccine for non-small cell lung cancer (NSCLC). L-BLP25 has shown improved survival rates for patients with NSCLC [72]. A phase III clinical trial is currently underway.

### 2.4. Immunostimulatory Complexes (ISCOMs)

Immunostimulatory complexes (ISCOMs) are particulate antigen delivery systems composed of antigens, cholesterol, phospholipids, and saponins [73]. ISCOMs are 40 nm cage-like particles used for entrapping hydrophobic antigens. ISCOMs can enhance the antigenic response in both oral and parenteral delivery. Studies have shown an enhanced immunogenic response when portions of the influenza virus and cholera toxin [74] were integrated into ISCOMs for delivery. Trudel et al. [75] were the first to introduce ISCOMs for the respiratory syncytial virus and found its capabilities of producing serum-neutralizing antibodies and T cells when given to mice [75]. Another similar delivery system, ISCOMATRIX, is composed of similar components, but it does not have the antigen. The antigen can be added to the ISCOMATRIX system separately during vaccine preparation [76]. With the addition of an in-built adjuvant, ISCOMs and ISCOMATRIXTM are superior carrier systems compared to conventional carrier systems. Moreover, they have also been proven to be more immunogenic than most particulate colloidal systems [77]. Studies for ISCOM flu vaccines have shown that a single dose enhanced influenza A virus-specific cytotoxic T Lymphocyte memory 10–12 times more compared to those of the standard influenza vaccine [78]. Studies have also been conducted for ISCOM/ISCOMATRIX vaccines for the human papillomavirus (HPV) [37], human immunodeficiency virus (HIV), herpes simplex virus (HSV), hepatitis C virus (HCV), and cancer [79]. These studies revealed both cellular and humoral immune responses without any significant safety concerns for humans [37,78]. ISCOMs have been extensively studied in animal models where they have been shown to induce strong immune responses [80,81]. Thus, ISCOM/ISCOMATRIX vaccines have been approved for veterinary purposes and use in many clinical trials for human use at present [82,83]. ISCOMs require a reduced number of antigens and adjuvants to induce immunity compared to vaccines made by mixing saponins and antigens [82]. However, certain ISCOMs have raised safety concerns for actual human use since some saponins are toxic for human use when used at high concentrations, although other saponins, such as QS-21 and QuilA, have not shown toxicity at administered doses [79].

### 2.5. Emulsions

Emulsions are another commonly used delivery platform in vaccine development (Table 1). Emulsions are a mixture of two or more immiscible liquids: either in a dispersed or continuous phase. For vaccine emulsions, there are two phases: antigenic media (usually in water) and oil. Different kinds of emulsions can be formulated for vaccine delivery such as water-in-oil emulsions, oil-in-water emulsions, and water-in-oil-in-water emulsions, as well as emulsions based on mineral oils and non-mineral oils. Water-in-oil emulsions incite powerful consistent immune responses, whereas oil-in-water emulsions induce a short-term immune response. On the other hand, water-in-oil-in-water emulsions induce long- and short-term immune responses with different antigens. Mineral oil emulsions are effective, but they result in local reactions with reactive antigens. In contrast, non-mineral oils are well tolerated but comparatively inefficient with poor immunogens. Adjuvant emulsions generate depots entrapping antigens at the injection site, thus resulting in a slow release of the antigens over a period of time. This causes a continuous stimulation of the immune system and enhances the activation of antigen-presenting cells (APCs). The most common oil-in-water emulsions licensed for vaccine development are MF59 and Freund’s complete adjuvant. MF59 causes stimulation of both cellular (Th1) and humoral (Th2) immune responses. MF59 prevents antigens from rapid degradation and creates inflammation to stimulate the antigens’ macrophages. Freund’s complete adjuvant creates depots at the injection site that release antigens over a period of time. A study by Vesikari et al. showed the effects of the O/W emulsion adjuvant with the influenza vaccine, where kids aged 6–72 months were given trivalent-inactivated influenza vaccine (TIV) with and without the MF59 adjuvant, and the control group was administered non-influenza vaccines. The results showed that the vaccine with MF59 was the most effective, with only 0.7% of the children catching influenza compared to 2.8% without the adjuvant, and 4.7% within the control group [84]. The choice of the emulsion depends on the target species, as some species react more to the vaccines than others. The complication of emulsion-based adjuvants lies in their likelihood of inducing autoimmunity.

### 2.6. Virus-like Particles and Virosomes

Virus-like particles (VLPs) are self-assembling nanostructures made of viral structural proteins. The infectious genetic material is removed from the virus, making it inert/non-pathogenic. A virosome is a type of “artificial virus” that can work as a delivery vehicle to deliver vaccine antigens directly into host cells [85]. Both VLPs and virosomes are capable of penetrating into the cells while maintaining structural integrity, and they can then induce both cellular as well as humoral immunity [86,87,88]. There are many advantages to using VLPs and virosomes in vaccine production. These vaccines are easy to produce, mostly have a good safety profile, and strongly stimulate the immune system, as well as being good for epicutaneous delivery, nasal delivery, and mucosal immunization. The most used virus vectors are adenoviral vectors from adenoviruses [89]. For example, RTS,S is an adenovirus recombinant malaria vaccine created by integrating the hepatitis B surface antigen into the plasmodium falciparum-derived circumsporozoite (CS) protein. The vaccine provides 56% protection against naturally occurring malaria infections [90].

Virosome-based vaccines, such as EpaxalTM, a hepatitis A vaccine, and Inflexal^®^ V, an influenza vaccine, are manufactured by Berna Biologics Ltd. [91,92]. Invivac^®^ is also a virosome-based flu vaccine in Switzerland and the Netherlands. The most recently approved VLP vaccine is Gardasil^®^ for immunization against the human papillomavirus (HPV). The vaccine has been shown to be 90% to 100% effective. The vaccine is also effective in preventing cervical cancer and genital warts [93]. VLP-based vaccines for many diseases such as the SARS coronavirus, Ebola virus, hepatitis C virus (HCV), food-borne norovirus infection, mosquito-borne chikungunya virus, influenza, malaria, rotavirus, etc., are currently in preclinical and clinical stages. It is highly likely that some of them will eventually obtain approval for human vaccination in the near future.

## 3. The Vaccine Development Approach for Coronavirus

For the development of vaccines against coronavirus, many approaches have been considered since the outbreak of COVID-19. A list of coronavirus vaccines currently in clinical trials in the USA is detailed in Table 2. In the first line of defense to generate a vaccine against COVID-19, traditional vaccine formulations using the entire virus (either as an attenuated live virus or inactivated/engineered virus), virus-like particles (VLPs), viral vectors (replicating and non-replicating), etc., and DNA, RNA, protein, etc., as antigens have been considered. However, finding the right approach to generate a COVID-19 vaccine cannot be a simple task, as it is known that many of these approaches could trigger immune responses against the host or exert an unwanted immune response [94,95]. Moreover, once the outbreak of coronavirus occurred in 2019, the demand for immediate treatment for COVID-19 viral infection, as well as for effective vaccination against this virus, was soaring. In response to this crisis, scientists, pharmaceutical and biotech companies, government health agencies, and more came together to find a way to control and minimize the outbreak.

No mRNA-based vaccine had been approved for human use before the COVID-19 pandemic [96]. The use of nucleic acids such as siRNA, mRNA, or pDNA for the treatment of infectious diseases, cancer, etc., was not new [97,98,99,100]. Also, the nanoformulations delivered nucleic acids for human use were approved far earlier before the coronavirus outbreak [101,102,103]. Amidst this COVID-19 outbreak, mRNA technology brought hope and relief to fight against COVID-19 [96]. BioNTech/Pfizer and Moderna, through the use of mRNA technology, have torn up conventional timelines of vaccine manufacturing and production, as they were able to produce trial vaccines for testing within weeks. These two companies were the first to obtain approval for using mRNA in vaccine production for human use.

Generally, an immune response in hosts against the COVID-19 virus can be achieved by injecting a small DNA or mRNA genetic sequence of the specific viral protein of the coronavirus via the nanotechnology platform. The most notably used viral proteins of the coronavirus are spike proteins, which are known to maintain a high conservation of their genetic sequences over time [104,105]. One important question to ask is to choose the right nucleic acid, either DNA or mRNA, to generate the immune response in hosts against COVID-19. mRNA-based therapies have been proven to have several advantages over DNA-based vaccines [106]. mRNA is not infectious, and, unlike DNA, it would not be integrated into the host genome. mRNA is generally short-lived, and it can be regulated by adding a certain capping sequence or by modifying secondary structures in the 5′ and 3′ untranslated regions for better ribosome accessibility [107]. Hence, unlike DNA (which needs the host nucleus for this DNA to be decoded into protein), mRNA is advantageous in that it does not need to cross another phospholipid bilayer of the nucleus in the host cells in addition to the host cell membrane. However, due to the presence of nucleases in both the blood serum and host cellular environment, mRNA needs to be shielded from the nucleases en route to the host cells. As such, mRNA needs a carrier that can safely and efficiently deliver mRNA cargo into the host cells.

As we discussed earlier regarding the choices of many different types of delivery vehicles (Table 1) to deliver nucleic acids to the host cells, for COVID-19, the vehicle of choice was lipid nanoparticles [108]. Due to the charge interactions, negatively charged mRNA can be easily complexed with the positively charged lipids, which will provide them stability and prevent them from RNase-mediated degradation while being delivered into the cells. Though other delivery vehicles, such as polymeric nanoparticles, inorganic nanoparticles, ISCOMs, emulsions, virus-like particles and virosomes, protein nanoparticles, etc. (summarized in Table 1), have been used for vaccination earlier, many of those delivery systems have not been assessed extensively for a COVID-19 vaccine. Polymeric nanoparticles are quite promising in vaccine and antibody delivery due to their characteristic structural flexibility and design. Chen et al. recently showed a coronavirus antigen-coated biopolymer particle (BP) that can induce protective immunity against COVID-19 [109]. Non-replicating adenovirus vectors have also been tried in an effort to develop a vaccine against COVID-19. For example, the adenovirus type 5 vector (Ad5-nCoV), as of 16 March 2020, and the chimpanzee adenovirus vaccine vector (ChAdOx1), as of 31 March 2020, by CanSino Biological and the University of Oxford, respectively, were among some of the adenovirus vectors that have been tried recently [110]. Though adenoviral vectors are favorable for their broad tissue tropism, scalability, and other such factors, a pre-existing immunity against some adenoviral vectors in humans has also been reported [111], which hampers the feasibility of using those for COVID-19 vaccination. On the other hand, the adjuvant has an important role in the efforts for COVID-19 vaccination, as it might induce heterotypic responses against different variants or strains of the same virus [112]. Yang et al. recently developed a protein-based vaccine BCVax, which is a nanoparticle-immune stimulation complex (AB801-ISCOM) consisting of the antigen delta strain spike protein and the QS21-based adjuvant AB801 (which produced high levels of the anti-S protein IgG after two doses of BCVax in animal models and was capable of neutralizing multiple variants of COVID-19, including omicron BA.1 and BA.2 strains [113]). Though ISCOMs are prominent delivery systems for antigens and adjuvants, the complicated preparation, as well as safety concerns of some ISCOMs for human use [114], pose some disadvantages in using ISCOMs for vaccine delivery.

As of June 2020, 157 vaccine candidates were under consideration for development by academic labs, as well as by the industry and their partners [115]. A summarized list of the delivery vehicles that have been currently tried for the delivery of coronavirus vaccines in clinical trials in the United States of America is documented in Table 2. However, the vehicle of choice for antigen delivery was found to be lipid nanoparticles. The choice of liposomes in both clinical trials and FDA-approved drugs lies in the fact that the liposomes show remarkable results due to their high bioavailability and relatively low immunogenicity [116,117,118]. In the middle of November 2020, when Moderna revealed the results of the phase 3 clinical trial of a COVID-19 vaccine preventing nearly 95% of virus infection (which was followed by a similar report published by BioNTech and Pfizer (on 18 November 2020)), the invention stirred curiosity and disbelief, while also bringing hope and optimism [96]. The Pfizer-BioNTech and Moderna COVID-19 vaccines were the first mRNA-based vaccines authorized for emergency use in several countries to combat the COVID-19 pandemic. These vaccines demonstrated high efficacy rates in clinical trials and played a pivotal role in the initial global vaccination efforts against COVID-19 [96,101,119]. Eventually, COVID-19 mRNA vaccines have proven a tremendous blessing in protecting human lives from COVID-19. According to the WHO, as of 27 September 2023, more than 70.0% of the world population has received at least one dose of a COVID-19 vaccine; a massive 13.5 billion doses of COVID-19 vaccines have been administered globally. The achievement of this massive number of COVID-19 vaccinations came as no surprise, as the real-time data from different study settings showed an astonishing 91.2% and 98.1% effectiveness for the Pfizer–BioNTech vaccine and the Moderna vaccine, respectively [120]. Furthermore, this was against a virus whose rise was considered uncontrollable and untreatable in the early days of the COVID-19 outbreak.

## 4. Conclusions

The development of vaccines in recent years has helped us understand their molecular and cellular mechanisms, as well as what could be conducted to improve them. Nanoparticle vaccines offer several advantages that include, but are not limited to, stimulating the immune system, shielding antigens from degradation, and aiding targeting and control release. However, certain disadvantages regarding the nanoparticle vaccines also apply, like having a high surface area, challenges in crossing the biological membrane, and high reactivity. With that being said, a novel nanoparticle vaccine needs to be safe and tolerable before being approved for use. There are many nanoparticle vaccine adjuvants as well as delivery systems for cancer, malaria, AIDS, hepatitis, etc., that are currently in clinical trials. The potential for these agents/delivery systems to be marketed for human use requires thorough learning and development. The success of mRNA vaccines for COVID-19 that was delivered by a lipid-based nanoparticle system and obtained approval for human use for the first time opens the window for an impactful application of nanomedicine for the treatment and protection of human lives from diseases at a global scale.

## Figures and Tables

**Table 1:** Nanocarriers for the delivery of vaccines.

Delivery System	Composition	Antigen	References
Polymeric-Based System	PLGA	OVA	Demento et al. [5]
	PLGA, polylactic acid	Hepatitis B surface antigen	Thomas et al. [6]
	Lipid-coated PLGA	OVA	Bershteyn et al. [7]
	Lipid-coated PLGA	Malaria antigen	Moon et al. [8]
	Deacylated cationic polyethyleneimine	HIV CN54gp140 antigen	Mann et al. [9]
	Polylactic acid	Hepatitis B surface antigen	Saini et al. [10]
	Chitosan-coated polycaprolactone	H1N1 hemagglutinin	Gupta et al. [11]
	Polyanhydrides	Yersinia pestis antigen	Ulery et al. [12]
	Chitosan nanoparticles	HBsAg	Lugade et al. [13]
	Mannosylated chitosan nanoparticles	Recombinant hepatitis B virus surface antigen	Mehrabi et al. [14]
	Cholesteryl-conjugated pullulan	Clostridium botulinum type-A neurotoxin subunit antigen	Nochi et al. [15]
	N-trimethyl chitosan	OVA	Slutter et al. [16]
	Alginate nanoparticles	Diphtheria toxoid	Sarei et al. [17]
	Hyaluronic acid (HA), monophosphoryl lipid A (MPLA), aluminum salt (Alum)	Hepatitis B antigen	Moon et al. [18]
Inorganic Nanoparticles	Gold nanoparticles	Escherichia coli-specific immunogenic antigens	Sanchez-Villamil et al. [19]
	Gold nanoparticles	West Nile virus envelops protein	Niikura et al. [20]
	Carbon nanoparticles	Bovine serum albumin	Wang et al. [21]
	Carbon magnetic nanoparticles	Hen egg lysozyme	Schreiber et al. [22]
	Mesoporous silica nanoparticles	Schitosoma mansoni	Montalvo-Quiros et al. [23]
	Silica nanoparticle-based drug delivery system	H1N1 influenza hemagglutinin antigen	Neuhaus et al. [24]
	Alum	Combination of an influenza antigen	Knudsen et al. [25]
	Calcium phosphate nanoparticle	H1N1 hemagglutinin antigen	Morcol et al. [26]
Liposomes	DOPC, DOPG, MPB	OVA	Moon et al. [27]
	EPC, DOGS-NTA-Ni	His-tagged heat shock protein	Mašek et al. [28]
	Pegylated DDA, TDB	Ag85B-ESAT-6	Kaur et al. [29]
	DDA, TDB	OVA	Milicic et al. [30]
	DDA, DSPC, cholesterol, TDB	Ag85B-ESAT-6	McNeil et al. [31]
	DDA, TDB	Trivalent influenza vaccine	Rosenkrands et al. [32]
	DDA, TDB	Ag85B-ESAT-6	Henriksen-Lacey et al. [33]
	DDA, DODA, TDB	Ag85B-ESAT-6	Christensen et al. [34]
	Lecithin, cholesterol	Diphtheria toxoid	de Veer et al. [35]
Immunostimulatory Complexes (ISCOMS)	Cholesterol, phospholipids, saponins	hemagglutinin antigen	Cox et al. [36]
	ISCOMATRIX	HPV16 E6 and E7 recombinant bacterial fusion protein	Frazer et al. [37]
Emulsion	MF59	Recombinant meningococcal B protein	Brito et al. [38]
	MF59	Hemagglutinin	Calabro et al. [39]
	MF59	Recombinant meningococcal B protein	Singh et al. [40]
	W805EC	OVA	Myc et al. [41]
	W805EC	OVA	Makidon et al. [42]
	GLA	Falciparum subunit	Lousada-Dietrich et al. [43]
	GLA-SE	Recombinant hemagglutinin	Treanor et al. [44]
	GLA-SE	Plasmodium vivax subunit	Lumsden et al. [45]
Virus-Like Particles And Virosomes	Epaxal® (Crucell, Leiden, The Netherlands) A (H1N1) virosomes + inactivated hepatitis A virus		Bovier et al. [46]
	Inflexal® V (Crucell) Virosomes from three influenza strains: A (H1N1), A (H3N2), and B		Herzog et al. [47]
	Nasalflu® (Berna Biotech, Bern, Switzerland) Virosomes from three influenza strains: A (H1N1), A (H3N2), and B + heat labile toxin adjuvant		Gluck et al. [48]; Mutsch et al. [49]
	Invivac® (Solvay, Brussels, Belgium) Virosomes from three influenza strains: A (H1N1), A (H3N2), and B		de Bruijn et al. [50]; de Bruijn et al. [51]
	Epaxal® Junior (Crucell) A (H1N1) virosomes + inactivated hepatitis A virus		Bovier et al. [46]; Van der Wielen et al. [52]

**Table 2:** Coronavirus vaccines currently in clinical trials in the United States of America.

Study Title	Clinical Trails Gov ID	Clinical Trial	Interventions
Training the Innate Immune System Against SARS-CoV-2 (COVID-19) Using the Shingrix Vaccine in Nursing Home Residents (NH-Shingrix)	NCT04523246	Early Phase 1	Biological: SHINGRIX (zoster vaccine Recombinant, adjuvanted) Drug: normal saline
A Study Assessing the Safety, Tolerability, Immunogenicity of COVID-19 Vaccine Candidate PRIME-2-CoV_Beta, Orf Virus Expressing SARS-CoV_2 Spike and Nucleocapsid Proteins	NCT05367843	Phase 1	Drug: PRIME-2-CoV_Beta
Phase 1 Study of Intranasal PIV5 COVID-19 Vaccine Expressing SARS-CoV-2 Spike Protein in Healthy Adults and Adolescents (CVXGA1-001)	NCT04954287	Phase 1	Biological: CVXGA1 low dose Biological: CVXGA1 high dose
Safety And Immunogenicity Of HDT-301 Targeting A SARS-CoV-2 Variant Spike Protein	NCT05132907	Phase 1	Biological: HDT-301
Delayed Heterologous SARS-CoV-2 Vaccine Dosing (Boost) After Receipt of EUA Vaccines	NCT04889209	Phase 1 Phase 2	Biological: Ad26.COV2.S Biological: BNT162b2 Biological: mRNA-1273 Biological: mRNA-1273.211 Biological: mRNA-1273.222 Biological: SARS-CoV-2 rS/M1
GLS-5310 Vaccine in Healthy Volunteers as a Booster for SARS-CoV-2 (COVID-19)	NCT05182567	Phase 1	Drug: GLS-5310 (Group 1) Drug: GLS-5310 (Group 2) Drug: GLS-5310 (Group 3) Drug: GLS-5310 (Group 4)
COVID-19 Variant Immunologic Landscape Trial (COVAIL Trial)	NCT05289037	Phase 1 Phase 2	Drug: AS03 Biological: BNT162b2 Biological: BNT162b2 (B.1.1.529) Biological: BNT162b2 (B.1.351) Biological: BNT162b2 bivalent (wild type and Omicron BA.1) Biological: BNT162b2 bivalent (wild type and Omicron BA.4/BA.5) Biological: CoV2 preS dTM [B.1.351] Biological: CoV2 preS dTM/D614 Biological: CoV2 preS dTM/D614 + B.1.351 Biological: mRNA-1273 Biological: mRNA-1273.351 Biological: mRNA-1273.529 Biological: mRNA-1273.617.2 Other: sodium chloride, 0.9%
A Safety, Reactogenicity, and Immunogenicity Study of mRNA-1045 (Influenza and Respiratory Syncytial Virus [RSV]) or mRNA-1230 (Influenza, RSV, and Severe Acute Respiratory Syndrome Coronavirus 2 [SARS-CoV-2]) Vaccine in Adults 50 to 75 Years Old	NCT05585632	Phase 1	Biological: mRNA-1010 Biological: mRNA-1345 Biological: mRNA-1273.214 Biological: mRNA-1045 Biological: mRNA-1230
Chimpanzee Adenovirus and Self-Amplifying mRNA Prime-Boost Prophylactic Vaccines Against SARS-CoV-2 in Healthy Adults	NCT04776317	Phase 1	Biological: ChAdV68-S Biological: ChAdV68-S-TCE Biological: SAM-LNP-S Biological: SAM-LNP-S-TCE Other: sodium chloride, 0.9%
A Live Recombinant Newcastle Disease Virus-vectored COVID-19 Vaccine Phase 1 Study	NCT05181709	Phase 1	Drug: sodium chloride Biological: NDV-HXP-S IN low dose Biological: NDV-HXP-S IM low dose Biological: NDV-HXP-S IN high dose Biological: NDV-HXP-S IM high dose
Safety and Immunogenicity Study of a Booster Dose of the Investigational CV0501 mRNA COVID-19 Vaccine in Adults at Least 18 Years Old	NCT05477186	Phase 1	Biological: CV0501 (3 μg) Biological: CV0501 (6 μg) Biological: CV0501 (12 μg) Biological: CV0501 (25 μg) Biological: CV0501 (50 μg) Biological: CV0501 (75 μg) Biological: CV0501 (100 μg) Biological: CV0501 (150 μg) Biological: CV0501 (200 μg)
SARS-CoV-2-Spike-Ferritin-Nanoparticle (SpFN) Vaccine With ALFQ Adjuvant for Prevention of COVID-19 in Healthy Adults	NCT04784767	Phase 1	Biological: 25 µg SpFN_1B-06-PL + ALFQ (QS21 adjuvant) Drug: sodium chloride, USP, for injection (0.9% NaCl) Biological: 50 µg SpFN_1B-06-PL + ALFQ (QS21 adjuvant)
A Study of Modified mRNA Vaccines in Healthy Adults	NCT05397223	Phase 1	Biological: mRNA-1273 Biological: mRNA-1010 Biological: mRNA-1345 Biological: FLUAD^®^ Biological: mRNA-1647
Study of Recombinant Protein Vaccines With Adjuvant as a Primary Series and as a Booster Dose Against COVID-19 in Adults 18 Years of Age and Older (VAT00002)	NCT04762680	Phase 2 Phase 3	Biological: SARS-CoV-2 recombinant protein vaccine Phase 2 Formulation 1 Biological: SARS-CoV-2 recombinant protein vaccine Phase 2 Formulation 2 Biological: SARS-CoV-2 recombinant protein vaccine Phase 2 Formulation 3 Biological: SARS-CoV-2 adjuvanted recombinant protein vaccine, monovalent (D614)-AS03, Dosage A Biological: SARS-CoV-2 adjuvanted recombinant protein vaccine, monovalent (B.1.351)-AS03 Biological: SARS-CoV-2 adjuvanted recombinant protein vaccine, monovalent (D614)-AS03, Dosage B Biological: SARS-CoV-2 adjuvanted recombinant protein vaccine, monovalent (B.1.351)-AS03 Alternative Exploratory Formulation 1 Biological: SARS-CoV-2 adjuvanted recombinant protein vaccine, monovalent (B.1.351)-AS03 Alternative Exploratory Formulation 2 Biological: SARS-CoV-2 adjuvanted recombinant protein vaccine, monovalent (B.1.351)-AS03 Alternative Exploratory Formulation 3 Biological: SARS-CoV-2 adjuvanted recombinant protein vaccine, monovalent (B.1.351)-AS03 Alternative Exploratory Formulation 4 Biological: SARS-CoV-2 adjuvanted recombinant protein vaccine, bivalent (D614 + B.1.351)-AS03
Study of a Recombinant Coronavirus-Like Particle COVID-19 Vaccine in Adults	NCT04636697	Phase 2 Phase 3	Drug: intramuscular injection Biological: intramuscular vaccine
A Ph 2 Trial With an Oral Tableted COVID-19 Vaccine	NCT05067933	Phase 2	Drug: VXA-CoV2-1.1-S Other: placebo tablets
Safety and Immunogenicity of RNA-based Vaccines Against SARS-CoV-2 Variants in Healthy Participants	NCT05004181	Phase 2	Biological: BNT162b2 Biological: BNT162b2 (B.1.1.7 + B.1.617.2) Biological: BNT162b2 (B.1.1.7) Biological: BNT162b2 (B.1.617.2) Biological: BNT162b2 (B.1.1.529) Other: observational
COVID-19 VAX Booster Dosing in Patients With Hematologic Malignancies	NCT05028374	Phase 2	Drug: A single “booster” dose of the Moderna mRNA COVID-19 vaccine
A Study to Evaluate Safety and Effectiveness of mRNA-1273 COVID-19 Vaccine in Healthy Children Between 6 Months of Age and Less Than 12 Years of Age	NCT04796896	Phase 2 Phase 3	Biological: mRNA-1273 Biological: placebo Biological: mRNA-1273.214
A Phase 1/2/3 Study to Evaluate the Safety, Tolerability, and Immunogenicity of an RNA Vaccine Candidate Against COVID-19 in Healthy Children	NCT04816643	Phase 2 Phase 3	Biological: biological/vaccine: BNT162b2 10mcg Biological: BNT162b2 20mcg Biological: BNT162b2 30mcg Other: placebo Biological: biological/vaccine: BNT162b2 3mcg
A Study to Evaluate the Immunogenicity and Safety of mRNA Vaccine Boosters for SARS-CoV-2 (COVID-19) Variants	NCT04927065	Phase 2 Phase 3	Biological: mRNA-1273.211 Biological: mRNA-1273 Biological: mRNA-1273.617.2 Biological: mRNA-1273.213 Biological: mRNA-1273.529 Biological: mRNA-1273.214 Biological: mRNA-1273.222 Biological: mRNA-1273.815 Biological: mRNA-1273.231
Study to Evaluate Safety, Tolerability & Immunogenicity of BNT162b2 in Immunocompromised Participants ≥2 Years	NCT04895982	Phase 2	Biological: BNT162b2
COVID-19 Booster Vaccine in Autoimmune Disease Non-Responders	NCT05000216	Phase 2	Biological: Moderna mRNA-1273 Biological: BNT162b2 Biological: Ad26.COV2.S Drug: continue IS (MMF or MPA) Drug: continue IS (MTX) Biological: continue IS (B cell depletion therapy) Biological: monovalent (B.1.351) CoV2 preS dTM-AS03 Drug: withhold IS (MMF or MPA) Drug: withhold IS (MTX) Drug: withhold IS (B cell depletion therapy) Biological: Moderna mRNA-1273, bivalent Biological: BNT162b2, bivalent
A Study to Learn About Two or More Vaccines That Are Put Together as One Shot Against Infectious Lung Illnesses, Including COVID-19 and Respiratory Syncytial Virus (RSV)	NCT05886777	Phase 2	Biological: combination (RSVpreF + BNTb162b2) Biological: bivalent BNT162b2 (original/Omi BA.4/BA.5) Biological: RSVpreF Biological: QIV Biological: normal saline placebo
Study of Monovalent and Bivalent Recombinant Protein Vaccines Against COVID-19 in Adults 18 Years of Age and Older (VAT00008)	NCT04904549	Phase 3	Biological: SARS-CoV-2 adjuvanted recombinant protein vaccine (monovalent D614) (primary series) Biological: SARS-CoV-2 adjuvanted recombinant protein vaccine (bivalent D614 + B.1.351) (primary series) Biological: placebo Biological: SARS-CoV-2 adjuvanted recombinant protein vaccine (monovalent B.1.351) (booster dose) ≥4 months after last vaccination Biological: SARS-CoV-2 adjuvanted recombinant protein vaccine (monovalent D614) (primary series) and SARS-CoV-2 adjuvanted recombinant protein vaccine (monovalent B.1.351) (booster dose) ≥4 months after last vaccination
Phase 3 Study of Novavax Vaccine(s) as Booster Dose After mRNA Vaccines	NCT05875701	Phase 3	Biological: NVX-CoV2373 Biological: SARS-CoV-2 rS antigen/Matrix-M adjuvant
A Study to Evaluate Safety and Immunogenicity of mRNA-1273 Vaccine to Prevent COVID-19 in Adult Organ Transplant Recipients and in Healthy Adult Participants	NCT04860297	Phase 3	Biological: mRNA-1273
A Study to Evaluate the Safety and Immunogenicity of the mRNA-1273.214 COVID-19 Vaccine in Healthy Children Between 6 Months to Less Than 6 Years of Age	NCT05436834	Phase 3	Biological: mRNA-1273.214
ABNCoV2 Vaccine in Adult Subjects Previously Vaccinated for SARS-CoV-2	NCT05329220	Phase 3	Biological: ABNCoV2 Biological: Comirnaty
A Study to Evaluate the Efficacy, Immune Response, and Safety of a COVID-19 Vaccine in Adults ≥18 Years With a Pediatric Expansion in Adolescents (12 to <18 Years) at Risk for SARS-CoV-2	NCT04611802	Phase 3	Biological: SARS-CoV-2 rS/Matrix-M1 adjuvant (initial vaccination period) Other: placebo (initial vaccination period) Biological: SARS-CoV-2 rS/Matrix-M1 adjuvant (crossover vaccination period) Other: placebo (crossover vaccination period) Biological: SARS-CoV-2 rS/Matrix-M1 adjuvant (booster vaccination) Biological: SARS-CoV-2 rS/Matrix-M1 adjuvant (second booster vaccination)
Safety and Immunogenicity of 9-valent Human Papillomavirus (9vHPV) Vaccine Coadministered With Messenger Ribonucleic Acid (mRNA)-1273 Severe Acute Respiratory Syndrome Coronavirus 2 (SARS-CoV-2) (COVID-19) Vaccine (V503-076)	NCT05119855	Phase 3	Biological: 9vHPV vaccine Biological: mRNA-1273 vaccine
Platform Trial to Compare Homologous Boost of Authorized COVID-19 Vaccines and Heterologous Boost With UB-612 Vaccine	NCT05293665	Phase 3	Biological: UB-612 Biological: BNT162b2 vaccine Biological: ChAdOx1-S vaccine Biological: Sinopharm BIBP
BCG Vaccine for Health Care Workers as Defense Against COVID 19 (BADAS)	NCT04348370	Phase 4	Biological: BCG vaccine Biological: placebo vaccine

Source: ClinicalTrials.gov (as of 15 August 2023).
